# Trophic niches, diversity and community composition of invertebrate top predators (Chilopoda) as affected by conversion of tropical lowland rainforest in Sumatra (Indonesia)

**DOI:** 10.1371/journal.pone.0180915

**Published:** 2017-08-01

**Authors:** Bernhard Klarner, Helge Winkelmann, Valentyna Krashevska, Mark Maraun, Rahayu Widyastuti, Stefan Scheu

**Affiliations:** 1 J.F. Blumenbach Institute of Zoology and Anthropology, Animal Ecology, University of Göttingen, Göttingen, Germany; 2 Institut Pertanian Bogor—IPB, Department of Soil Sciences and Land Resources, Bogor, Indonesia; 3 Centre of Biodiversity and Sustainable Land Use, Göttingen, Germany; University of Fribourg, SWITZERLAND

## Abstract

Conversion of tropical rainforests into plantations fundamentally alters ecological niches of animal species. Generalist predators such as centipedes (Chilopoda) may be able to persist in converted ecosystems due to their ability to adapt and switch to alternative prey populations. We investigated variations in community composition and trophic niches of soil and litter living centipedes in a range of ecosystems including rainforests, jungle rubber agroforests, and rubber and oil palm monocultures in two landscapes in Sumatra, Indonesia. Including information on environmental factors in the soil and litter habitat, we explored drivers shaping ecological niches of soil living invertebrate predators in one of the world’s hotspots of rainforest conversion. Conversion of rainforests into agroforests and plantations was associated with a marked change in the composition of centipede communities. However, irrespective of major differences in habitat characteristics, changes in total abundances were small and the overall diversity and biomass of centipedes was similar in each of the systems investigated, suggesting that the number of ecological niches for this group of predators remains unchanged. By using stable isotope analysis (^15^N and ^13^C), we investigated trophic niche shifts of the centipede community; lower δ^13^C values of centipedes in oil palm plantations as compared to other ecosystems suggests that centipedes switch from decomposer prey to other prey, presumably understory associated herbivores, due to reduced availability of litter associated prey species. The results suggest that the ability to utilize alternative prey is a key feature enabling invertebrate predators to persist in ecosystems undergoing major structural changes due to anthropogenic land use change.

## 1. Introduction

Large areas of tropical forest are logged and converted into cropland, driven by the global demand for commodities such as timber, biofuels and agricultural goods [1]. As a consequence, structurally complex habitats with species rich natural plant and animal communities are progressingly shrinking and making way for landscapes dominated by comparatively monotonous plantations. The intensive management of plantation systems is associated with disturbances such as soil degradation, which have been shown to influence soil animal diversity and biomass, thereby altering and reducing ecosystem services provided by these organisms [2–4]. In South-East Asia logged rainforest sites have been planted in large with rubber (*Hevea brasiliensis*) and oil palm (*Elaeis guineensis*), with in particular oil palm gaining importance in the last decades [5,6]. The world’s leading producer country for palm oil and the second ranking producer of natural rubber is Indonesia [7,8]. The Jambi province in Central Sumatra currently is in focus of a large-scale collaborative research project, investigating the ecological functions of natural rainforest and its transformation systems, as well as the human dimensions and economic factors driving the land use change (EFForTS—Ecological and socio-economic functions of tropical lowland rainforest transformation systems; for details see [9]). Sumatra underwent drastic deforestation in the last decades, with only 30% of its former forests remaining in 2007 [10]. At present the study region is shaped by small-holder and industrial scale rubber and oil palm plantations [11], but also by remnants of lowland rainforest, thereby providing the opportunity to study consequences of rainforest conversion into agroforestry / agricultural systems in a replicated design.

At the same sites investigated in the present study, key environmental processes have been measured and evaluated in detail, including soil organic carbon stocks and soil erosion [12], carbon pools and primary production in above- and belowground tree biomass [13], soil-atmosphere carbon dioxide and methane fluxes [14] and nitrogen cycling and soil fertility [15]. Further, selected groups of soil biota and their functional role were investigated, providing detailed data on root vitality, root stoichiometry and colonization of roots by mycorrhizae [16], the composition of the microbial communities [17,18] and the biodiversity, abundance and biomass of surface active terrestrial invertebrates [19]. Overall, the results document changes in soil properties and a substantial loss of biodiversity and ecosystem functions with intensified human land use in the study region [20]. Comparable tradeoffs between biodiversity and ecosystem functioning versus human land use are documented from other converted rainforest systems in Indonesia, e.g., cocoa agroforests in Sulawesi [21]. However, to date, the connections between individual organism groups and specific ecosystem functions, and how these are affected by the management of agricultural / agroforestry replacement systems, are little understood. Better knowledge on the functional role of individual organism groups is crucial for a successful development of strategies for retaining services provided by the soil microbial and animal community of former rainforest.

The aim of the present study was to gain insight into how invertebrate top predators adjust to anthropogenic impacts and conversion of their natural habitat by investigating and comparing the species composition and dietary niches of centipede (Chilopoda) communities in tropical rainforests, agroforests and plantations. Centipedes are an ubiquitous component of the terrestrial fauna in the tropics as well as in temperate regions. Most centipedes are generalist predators feeding on a broad spectrum of soil invertebrates, including Collembola, Diptera larvae and Lumbricidae [22–24]; diets of large Scolopendromorpha may also include small vertebrate prey [23,25]. Such generalist predators affect important ecosystem functions by regulating decomposer populations [26] and function as antagonists of agricultural pest species [27]. Centipedes are especially abundant in soil and litter of forests and agroforestry systems; in temperate beech forests centipede biomass typically exceeds that of other predatory arthropod groups [28]. Despite being key players in most terrestrial food webs, there is little knowledge on the ecology and functioning of centipedes and this applies in particular to tropical ecosystems.

Investigating variations in natural stable isotope ratios provides a powerful tool for assessing ecological niches of consumer species [29] allowing insight into the diet of organisms and the shift in their dietary niches with land use. In particular, natural variations in carbon and nitrogen stable isotope ratios provide complementary information for characterizing dietary niches of soil organisms [30]. Due to fractionation processes the body tissue of animals feature higher ^15^N/^14^N ratios compared to ratios of their diet [31–33]. The concentration of the heavy nitrogen isotope (^15^N) increases on average by 3.4 ‰, ^15^N/^14^N ratios therefore allow insight into the trophic level of species [34,35]. The fractionation of the heavy carbon isotope (^13^C) is much lower, averaging 0.4 ‰ [34], and therefore allows tracing basal food resources [32,34,36].

We investigated changes in centipede species composition along a gradient of increasing land use intensity, ranging from secondary rainforest to jungle rubber agroforests to intensive rubber and oil palm plantations. Further, we analyzed natural variations in ^15^N/^14^N and ^13^C/^12^C ratios of centipede species to characterize their trophic niches and investigate potential niche shifts with rainforest conversion. We related changes in trophic structure and community composition of centipedes to factors characterizing the basis of the soil food web, such as leaf litter deposition, C-to-N ratio and microorganisms of leaf litter and soil.

We hypothesized that (1) centipede abundance, biomass and species richness decrease from rainforest to jungle rubber to rubber and oil palm monocultures due to increasing disturbances by management practices. Further, we hypothesized that (2) stable isotope values (^15^N, ^13^C) of centipede species differ between rainforest and converted systems, reflecting changes in prey populations due to altered environmental conditions and changes in the availability and quality of prey with rainforest conversion.

## 2. Materials and methods

### 2.1. Study sites

We investigated four lowland rainforest systems in each of two study regions, i.e. Bukit Duabelas- (2° 0' 57" S, 102° 45’ 12" E) and Harapan landscape (1° 55' 40" S, 103° 15' 33" E), located in the Jambi province of southwest Sumatra, Indonesia. The four systems, rainforest, jungle rubber, rubber and oil palm, represent stages along a gradient of land-use intensity. Each system was replicated four times in each of the two landscapes resulting in a total of 32 sites. Rainforest sites used as reference were secondary rainforest stands close to natural conditions that underwent selective logging some 20–30 years ago. Jungle rubber agroforests originated from rainforests enriched with rubber trees (*Hevea brasiliensis*) but still containing remnants of the former rainforest vegetation; they were taken to represent rainforest conversion systems of low land use intensity as they lack inputs such as fertilization and herbicide application. Rubber and oil palm (*Elaeis guineensis*) mono-cultures were intensively managed plantations with an average age of 13 and 14 years, respectively, and represented high intensity systems. Soils at the study sites were acrisols with a sandy loam texture in Harapan and clay texture in Bukit Duabelas [12]. All study sites were on similar altitude varying between 50 and 100 m a.s.l. [12]. More details are given in [9].

### 2.2. Field work permissions

The study was conducted in the framework of the German—Indonesian research project “Ecological and socio-economic functions of tropical lowland rainforest transformation systems” (EFForTS) and is based on the research permits no. 332/SIP/FRP/SM/IX/2012, 389/SIP/FRP/SM/X/2013 and 145/SIP/FRP/SM/V/2013 issued by the State Ministry of Research and Technology of the Republic of Indonesia (RISTEK). Samples collected were based on collection permit no. S.07/KKH-2/2013 issued by the Indonesian Ministry of Forestry (PHKA). Sample exportation for analysis in Germany was supported by the Indonesian Institute of Sciences LIPI (register file no. 24/SI/MZB/IV/2014) and based on permit no. 125/KKH-5/TRP/2014 issued by Ministry of Forestry of the Republic of Indonesia.

### 2.3. Sampling, extraction and determination

Between October and November 2013 soil samples were taken from plots established at each study site. Each plot measured 50 x 50 m and featured five 5 x 5 m subplots distributed in a fixed arrangement at each site [9]. For the present study three samples were taken per study site each from a different subplot, resulting in a total of 96 samples. Samples were taken at random from the subplot at least 2 m distant to the next tree or accumulation of dead wood using a spade. Each sample measured 16 x 16 cm and included the full litter layer and underlying top soil to a depth of 5 cm. Soil animals were extracted by heat [37], material of the litter and topsoil layer (0–5 cm depth) was extracted separately. Centipedes were determined to species or morphospecies level and stored in 70% ethanol until further processing.

### 2.4. Estimation of population biomass

Individual body mass (dry mass) was either weighed or determined by body size—weight regressions. For the regressions body lengths (head to end of last body segment) and body widths (at the broadest body segment) of all specimens were measured using a camera equipped dissection microscope (Zeiss Stemi 2000-CS with Zeiss AxioCam ICc1) and the imaging software AxioVision LE64 Version 4.9.1.0. Specimens to be used for stable isotope analyses were dried and weighed (see section 2.5). Power law models were used to establish size—weight relationships of the form y = a (x)^b^, with y the dry weight (mg) and x the body size. To account for group specific differences of the body shape, separate regressions were calculated for long and slender species (47 Geophilomorpha specimens) and comparatively short and robust animals (16 Cryptopidae and Henicopidae specimens). For both groups three body size parameters were investigated, i.e. body length (mm), horizontal cross sectional area (body length × body width; mm^2^) and volume (assuming a cylindrical body shape; mm^3^). Horizontal cross sectional area correlated best with animal dry weight (S1 Table, S1 Fig) and therefore was used for all body size—weight regressions. The population biomass for each site was calculated as total dry weight of the individuals of the three samples expressed on a square meter basis. Scolopendridae were excluded from the analyses as only two specimens were caught.

### 2.5. Stable isotopes and C-to-N ratios

Centipede specimens, litter and soil material were dried at 60°C for 24 h. For measuring stable isotope ratios whole centipede specimens were used whenever possible; large animals exceeding the limits of the analysis system were homogenized with a mortar and pestle and an aliquot was used for measuring stable isotope values. Replicates from different sites were analyzed for each species whenever possible. Before measurement litter and soil material was dried and ground with a ball mill (Retsch Mixer Mill MM200, Haan, Germany). Stable isotope ratios, carbon and nitrogen concentrations of animals and of litter and soil material were determined using a coupled system of an elemental analyzer (NA 1500, Carlo Erba, Milan, Italy) and a mass spectrometer (MAT 251, Finnigan, Bremen, Germany) [38]. Isotopic values were expressed using the δ notation with δX (‰) = (Rsample−R_standard_) / R_standard_ x 1000, with X representing the target isotope (^15^N or ^13^C) and R the ratio of the heavy to the light isotope (^13^C/^12^C and ^15^N/^14^N, respectively). Nitrogen in atmospheric air and Vienna PD Belemnite served as standard for ^15^N and ^13^C, respectively, and acetanilide for internal calibration. Stable isotope values of animals are given as difference to the values of leaf litter sampled from the study sites, i.e. by calculating Δ^13^C and Δ^15^N values [39].

### 2.6. Environmental factors

Amount of litter at the study sites was measured by weighing the remaining dry litter material after extraction of soil animals and removal of inorganic material, large seeds and coarse woody debris. Soil and litter pH were measured in CaCl_2_ solution using a digital pH meter. Microbial biomass in litter and soil was determined by measuring O_2_ consumption using an automated respirometer system [40,41]; the data have been reported in detail elsewhere [18].

### 2.7. Data analysis

Differences between number of species, abundance and population biomass of centipedes in different conversion systems were inspected by linear mixed-effects models with landscape (Harapan and Bukit Duabelas) treated as random factor [42] in R 3.2.1 [43]. Hypothesis 1, i.e. that effects of conversion followed a linear trend (in the following referred to as “intensity hypothesis”), was investigated by defining a linear contrast for the conversion systems (of the order rainforest, jungle rubber, rubber and oil palm). In addition, planned comparisons were performed testing differences between each combination of two conversion systems. Abundance and population biomass were log_10_-transformed to meet assumptions of normality and modelled on a Gaussian distribution. Number of species was modelled on a Poisson distribution. Additionally, the influence of spatial autocorrelation was investigated by determining Moran’s I standard deviate [44] for the residuals of each model using package “spdep” [45] in R. The analysis provided no support for significant spatial autocorrelation, with all p-values > 0.05.

The total species pool for the sites investigated was determined using the function “specpool” of package “vegan” in R [46]. Discriminant function analysis was performed and squared Mahalanobis distances (MD^2^) determined to evaluate effects of forest conversion on the species composition of centipede communities using STATISTICA 7 (StatSoft Inc. 2005). Canonical Correspondence Analysis (CCA) was used to correlate species abundances and environmental factors structuring the centipede communities in the landscapes and conversion systems in CANOCO 5.02 (Ter Braak and Šmilauer 2012); landscapes and conversion systems were coded as supplementary variables.

Variations in the trophic position of centipede species, as indicated by Δ^13^C and Δ^15^N values, in different conversion systems were inspected using generalized linear mixed-effects models in R. Planned comparisons were performed testing differences between each combination of two conversion systems. Due to the data including cases of measurements from different specimens collected on the same plot, plot was included as random factor nested in landscape. The data were modeled on a Gaussian distribution.

## 3. Results

### 3.1. Centipede community structure

Twelve species of centipedes occurred at the study sites (Table 1), extrapolating the total species pool of all sites resulted in 12 (Chao estimation) and 13 species (first order jackknife and bootstrap estimation) (S2 Fig). Abundances differed significantly between rainforest and rubber plantations (F_1, 30_ = 5.75, p = 0.023), with values decreasing from 108.9 (SD = 74.5) to 40.6 (SD = 27.3) ind. /m^2^. Other pairwise comparisons of conversion systems were non-significant (values for each conversion system are given in Table 2, statistical results are given in S2 Table). Species number and biomass of centipedes did not differ significantly between conversion systems (S2 Table). The models investigating linear trends (intensity hypothesis) were non-significant for abundance, biomass and species number (S2 Table).

**Table 1:** List of centipede species including full taxonomic name and authority, family affiliation, number of replicates (stable isotope ratios) and mean Δ^13^C and Δ^15^N values (± SD) (see text for details).

Abbr.	Full name	Family	N	Δ^13^C	Δ^15^N
Cryp_sp	*Cryptops sp*. Leach, 1814	Cryptopidae	14	4.71 ± 0.79	6.54 ± 1.79
Lam_sp	*Lamyctes sp*. Meinert, 1868	Henicopidae	3	4.61 ± 1.05	5.67 ± 0.9
Mec_eni	*Mecistocephalus* cf. *enigmus* Chamberlin, 1944	Mecistocephalidae	2	5.48 ± 0.98	7.65 ± 2.73
Mec_ste	*Mecistocephalus* cf. *stenoceps* Chamberlin, 1944	Mecistocephalidae	28	4.79 ± 1.18	7.62 ± 1.25
Mec_un	undetermined Mecistocephalidae	Mecistocephalidae	0	NA	NA
Mec_ver	*Mecistocephalus* cf. *verrucosus* Verhoeff, *1937*	Mecistocephalidae	2	5.1 ± 0.52	9.41 ± 0.43
Par_sp	*Paracryptops sp*. Silvestri, 1924	Cryptopidae	0	NA	NA
Sche_sp	undetermined Schendylidae	Schendylidae	3	2.82 ± 0.64	5.69 ± 0.75
Scol_sp	undetermined Scolopendridae	Scolopendridae	1	3.25	6.99
Sun_bid	*Sundageophilus bidentatus* Bonato, 2016	Geophilidae	6	5.62 ± 1.15	7.72 ± 0.57
Sun_por	*Sundageophilus poriger* Bonato, 2016	Geophilidae	5	3.82 ± 0.76	9.14 ± 1.42
Tyg_jav	*Tygarrup* cf. *javanicus* Attems, 1929	Mecistocephalidae	1	4.32	5.66

Abbr., abbreviation used in figures; NA, not available.

**Table 2:** Mean species number, abundance and biomass (± SD) of centipedes in different rainforest conversion systems.

Conversion system	Species number	Abundance [ind. / m^2^]	Biomass [mg / m^2^]
Rainforest	3.13 ± 1.36	108.88 ± 74.5	31.62 ± 27.14
Jungle rubber	2.75 ± 1.49	78.00 ± 49.14	25.27 ± 33.67
Rubber	2.00 ± 1.20	40.63 ± 27.30	18.67 ± 32.68
Oil palm	2.25 ± 0.71	71.50 ± 37.42	20.74 ± 14.27

Discriminant function analysis indicated that the species composition of centipedes differed significantly between conversion systems (Wilks' λ = 0.13, F_30,56_ = 1.90, p = 0.02), with rainforest communities being distinct from those in jungle rubber (MD^2^ = 14.34, F_10,19_ = 3.89, p = 0.005), rubber (MD^2^ = 14.38, F_10,19_ = 3.90, p = 0.005) and oil palm (MD^2^ = 10.09, F_10,19_ = 2.74, p = 0.03; S3 Fig).

**Fig 1:**
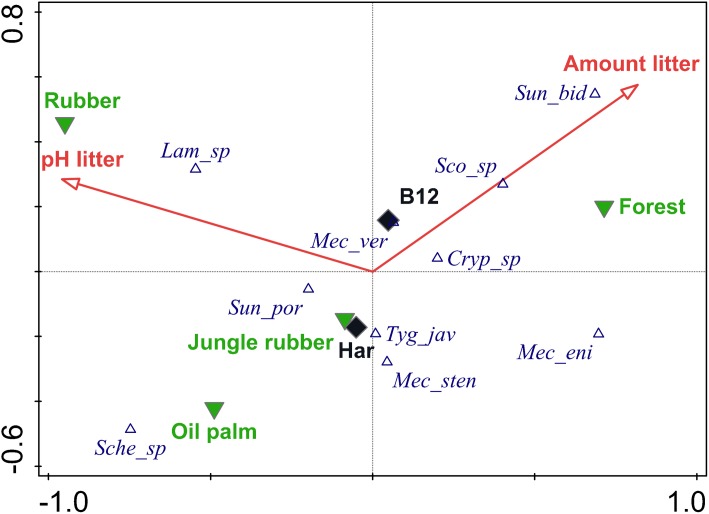
Canonical Correspondence Analysis (CCA) of centipede species in rainforest conversion systems of two study regions. Different symbols indicate centroids of species (empty blue triangles, full names are given in Table 1), conversion systems (filled green triangles) and study regions (filled black diamonds). Environmental factors used as constraints (red arrows) were amount of litter in the litter layer and pH of litter. Axis 1 accounted for 10.1% and Axis 1 and 2 cumulatively accounted for 16.2% of the variability in species data (Pseudo F = 3.1, p = 0.04 and Pseudo F = 2.7, p = 0.01, for axis 1 and all axes, respectively).

Canonical correspondence analysis using the full set of environmental variables indicated that species abundances were significantly correlated with litter pH and amount of leaf litter (pseudo F = 3.1, p = 0.004 and pseudo F = 2.8, p = 0.048, respectively), with significant conditional effects for litter pH only (pseudo F = 3.1, p = 0.002) (full CCA results are given in S3 Table, S4 Table). After reducing the set of constraining variables to litter pH and amount of litter, 16.2% of the variation in species data could be explained and fitted on two axes (Fig 1). Litter pH accounted for 9.8% and amount of litter for 6.4% of the variation, respectively. Centroids of the conversion systems were separated along the first axis (10.1% explained variation) in the order rubber, oil palm, jungle rubber and rainforest. Rubber plantations correlated with increasing litter pH, rainforests with increasing amounts of litter. On the second axis oil palm plantations were separated from rubber plantations and rainforests in the opposite direction of amount of litter. Centroids for the two landscapes, Bukit Duabelas and Harapan, were close to the center of the ordination suggesting that centipede communities in both were similar. The species *Mecistocephalus* cf. *stenoceps* and *Cryptops* sp. occurred across regions and conversion systems and therefore were positioned in the center of the ordination. *Lamyctes* sp. also occurred in each of the conversion systems, but was especially common in rubber plantations. *Sundageophilus bidentatus* occurred predominantly in rainforest, whereas *Sundageophilus poriger* was most abundant in jungle rubber. Other species were rare and occurred in few sites only; the abundance of species at each of the study sites is given in S5 Table.

### 3.2. Trophic niches

**Fig 2:**
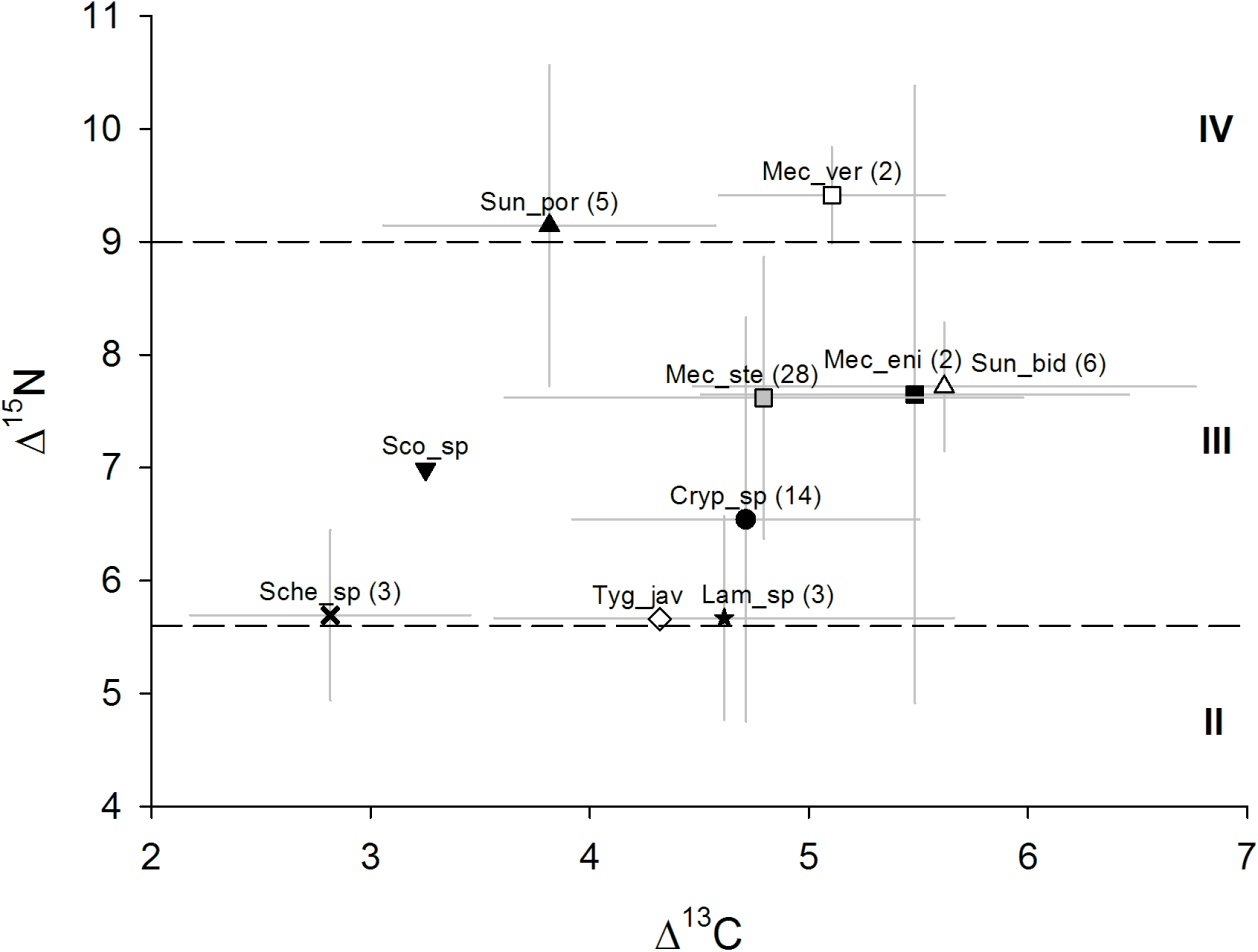
Mean (± SD) stable isotope values of centipede species. Data are normalized to the value of plant litter of the respective sampling site. Dashed horizontal lines represent estimated trophic level boundaries based on the mean leaf litter δ^15^N value, primary decomposers were assumed to constitute the first trophic level with mean values of approximately 0.5‰ above those of leaf litter [71], each trophic level was assumed to span 3.4‰ [34,35,72]; II = secondary decomposers and first order predators, III = second order predators, IV = third order predators. Numbers in brackets indicate the number of replicates; for full species names see Table 1.

Δ^13^C and Δ^15^N values varied between species but also between individuals of the species investigated (Fig 2). Both, the overall highest and lowest Δ^13^C value were measured for specimens of *M*. cf. *stenoceps*, with 1.88 ‰ and 7.17 ‰, respectively. Δ^15^N ranged from 2.85 ‰ in *Cryptops* sp. to 10.53 ‰ in *M*. cf. *stenoceps*. Means and standard deviation of Δ^13^C and Δ^15^N values for each species are given in Table 1, non-normalized δ^13^C and δ^15^N values of each species are given in S6 Table. Means and standard deviation of litter δ^13^C and δ^15^N values are provided in S7 Table.

**Fig 3:**
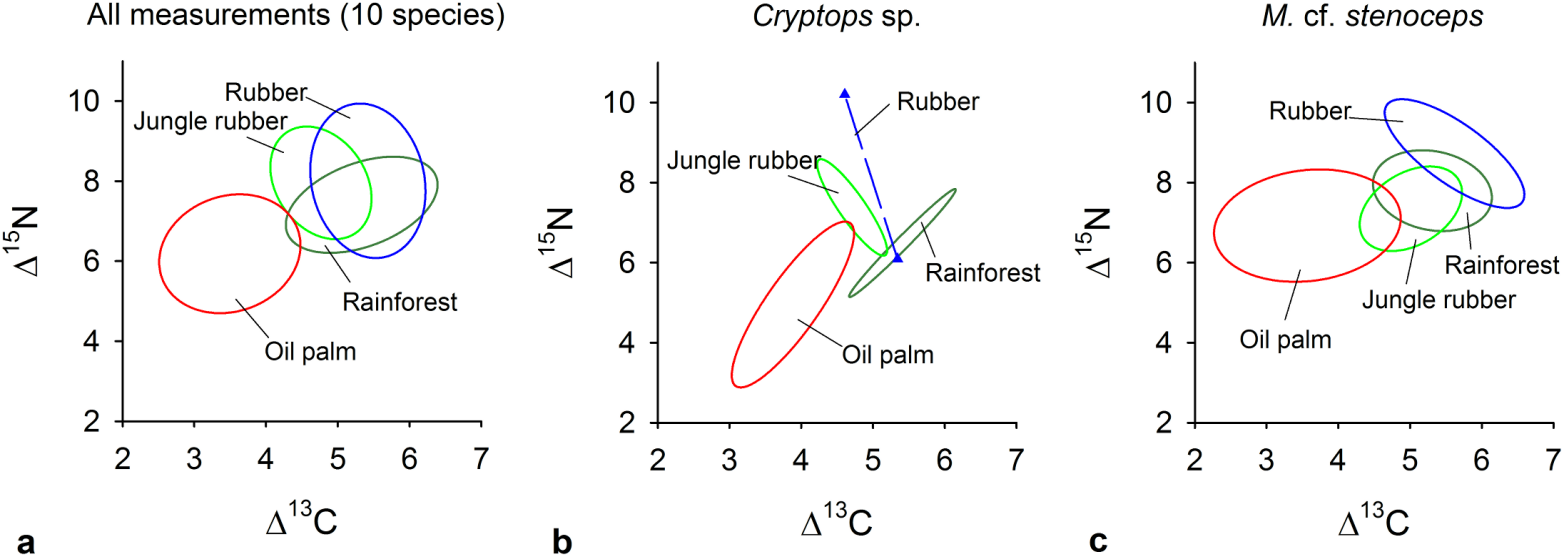
Isotopic niches of centipedes as affected by land use. Colored lines represent sample size corrected standard ellipse areas (SEAc [73]) of dual stable isotope values (δ^13^C, δ^15^N) of specimens collected in rainforest, jungle rubber, rubber and oil palm plantations using (a) the full set of measurements of 10 species (see also Table 1), (b) *Cryptops* sp. and (c) *Mecistocephalus* cf. *stenoceps*. Data are normalized to the values of plant litter collected at the respective sampling site.

Centipede stable isotope values differed significantly between conversion systems (Fig 3, statistical results are given in Table 3, values for each conversion system in Table 4). When investigating the full dataset of isotope values of centipede species, Δ^13^C values were significantly lower in oil palm plantations as compared to rainforests, jungle rubber- and rubber plantations; Δ^15^N values were lower in oil palm as compared to jungle rubber and rubber (Fig 3A). Values of *Cryptops* sp. in oil palm were significantly lower as compared to those from rainforest; Δ^15^N values of *Cryptops* sp. did not differ significantly between conversion systems (Fig 3B). In *M*. cf. *stenoceps* Δ^13^C values in oil palm were lower as compared to rainforest, jungle rubber and rubber; Δ^15^N values were lower in oil palm as compared to rubber (Fig 3C).

**Table 3:** ANOVA results from generalized linear mixed effects models testing the effect of forest conversion on stable isotope values of centipedes.

Contrast	All data		*Cryptops* sp.		*M*. cf. *stenoceps*	
	Δ^13^C	Δ^15^N	Δ^13^C	Δ^15^N	Δ^13^C	Δ^15^N
F vs. J	F_1, 22.9_ = 1.07	F_1, 18.0_ = 0.42	F_1, 9.5_ = 2.59	F_1, 9.3_ = 0.58	F_1, 16.7_ = 0.21	F_1, 13.5_ = 0.15
F vs. R	F_1, 25.9_ = 0.15	F_1, 22.8_ = 1.04	F_1, 10.5_ = 1.4	F_1, 10.5_ = 2.27	F_1, 17.3_ = 0.01	F_1, 15.8_ = 1.05
F vs. O	**F**_**1, 23.3**_ = 14.77***	F_1, 20.4_ = 2.56	**F**_**1, 9.5**_ = 12.84**	F_1, 10.0_ = 0.42	**F**_**1, 17.1**_ = 8.11*	F_1, 17.0_ = 2.95
J vs. R	F_1, 24.7_ = 0.45	F_1, 20.8_ = 0.15	F_1, 10.0_ = 0.64	F_1, 10.1_ = 0.21	F_1, 16.3_ = 0.32	F_1, 4.9_ = 3.00
J vs. O	**F**_**1, 23.3**_ = 5.86*	**F**_**1, 14.1**_ = 7.85*	F_1, 9.5_ = 1.09	F_1, 9.5_ = 1.09	**F**_**1, 16.0**_ = 5.25*	F_1, 15.8_ = 1.68
R vs. O	**F**_**1, 24.5**_ = 12.87**	**F**_**1, 23.9**_ = 9.46**	F_1, 9.7_ = 3.52	F_1, 9.6_ = 3.39	**F**_**1, 15.2**_ = 11.02**	**F**_**1, 25.2**_ = 10.71**

Results are given for planned comparisons between conversion systems (F = rainforest, J = jungle rubber-, R = rubber- and O = oil palm plantations) using the full set of measurements of 10 species (see also Table 1), data of *Cryptops* sp. and *Mecistocephalus* cf. *stenoceps*. Data are normalized to the values of plant litter collected at the respective sampling site. Degrees of freedom given represent Satterthwaite approximations.

* p < 0.05

** p < 0.01

*** p < 0.001.

**Table 4:** Mean Δ^13^C and Δ^15^N values (means Δ SD) of centipedes in different rainforest conversion systems.

Conversion system	all Chilopoda		*M*. cf. *stenoceps*		*Cryptops sp*.	
	Δ^13^C	Δ^15^N	Δ^13^C	Δ^15^N	Δ^13^C	Δ^15^N
Rainforest	5.33 ± 1.02	7.41 ± 1.16	5.31 ± 0.74	7.80 ± 0.90	5.41 ± 0.61	6.49 ± 1.10
Jungle rubber	4.77 ± 0.69	7.96 ± 1.37	5.01 ± 0.66	7.35 ± 0.98	4.71 ± 0.40	7.38 ± 0.98
Rubber	5.42 ± 0.76	8.01 ± 1.83	5.62 ± 0.87	8.72 ± 1.22	4.98 ± 0.52	8.15 ± 2.91
Oil palm	3.50 ± 0.95	6.19 ± 1.44	3.57 ± 1.20	6.92 ± 1.30	3.88 ± 0.70	4.95 ± 1.69

## 4. Discussion

### 4.1. Effects of rainforest conversion on centipede community structure

Overall, twelve centipede species occurred at the study sites; most species were sampled at least twice and extrapolations indicate that our sampling covers the spectrum of soil and litter living centipede species at the study sites. Mean abundances of centipedes were in the range of values reported from Malaysian rainforests [47] and Amazonian upland forests [48]. When compared to numbers reported by studies from Central European beech forests using similar sampling methods [28,49], the overall species number was similar, but abundances and especially biomasses were much lower, the latter approximately by a factor of ten. Abundances of other major soil arthropod groups, such as Collembola, Oribatida and Mesostigmata, were also low in comparison to Central European beech forests (B. Klarner, A. Potapov, D. Sandmann, S. Scheu, unpubl. data). This indicates that comparatively little energy is channeled to higher trophic levels in tropical decomposer food webs as suggested recently [19] and supports the view that tropical decomposer communities suffer from resource shortage caused by a combination of poor quality resources and high energy demand at high ambient temperatures [50,51]. Competition among predators as well as intra-guild predation may also differ in tropical as compared to temperate forest communities and result in low centipede numbers and biomass. Ants were the most abundant macroarthropods at the study sites [52] and function as predators in soil food webs [53]. Ants have been shown to exert direct and indirect effects on other predators [54,55]. However, in our samples abundances of ants and centipedes were positively correlated although the correlation was not significant (r = 0.31, p = 0.081). Nevertheless, this indicates that competition or other antagonistic interactions between ants and centipedes are of little importance. Spatial separation may have contributed to the lack of antagonistic interactions as most centipede species at the study sites were Geophilomorpha, living and hunting deeper in soil, whereas ants typically forage in the litter layer or above the ground.

Some centipede species, i.e. *M*. cf. *stenoceps*, *Cryptops* sp. and *Lamyctes* sp. occurred at a large number of sites suggesting that they are habitat generalists. Other species were rare and restricted to certain sites, suggesting narrower ecological niches and/or a preference for more patchily distributed microhabitats. The environmental variables measured indicate that changes in centipede communities with conversion of rainforest are driven by increasing pH in the litter layer, which is caused by fertilization [15]. An additional driver is the reduction of litter layers which was most pronounced between rainforests and oil palm plantations. Both factors likely affect centipedes via influencing abundances of prey taxa. A number of potential prey taxa are pH sensitive; in particular earthworm and enchytraeid species are known to preferentially occur at higher soil pH [56,57], and likely are an important prey for soil and litter centipedes [23,24]. The thickness of the litter layer is known to influence the composition of soil communities due to litter functioning as resource for decomposer species, forming the basis of forest soil food webs [58,59]. Thick litter layers additionally provide habitat structure and regulate abiotic conditions [60–62], which likely beneficially affect centipedes as well as other soil animal taxa.

We found negative effects of rubber cultivation on centipedes, with rubber plantations sustaining lower abundances compared to rainforest. Centipede abundance as well as diversity and biomass in jungle rubber and oil palm also was lower than in rainforest, however, the differences were not significant suggesting that centipedes are more resistant against conversion of rainforest into jungle rubber and oil palm as compared to conversion to rubber plantations. Potentially, increased sampling effort and higher statistical power would have allowed detecting more subtle changes in centipede diversity, abundance and biomass associated with rainforest conversion. Nevertheless, the results of our study indicate that rainforest conversion systems may sustain well developed centipede communities. This extends the findings of Barnes et al. [19], who found substantial decrease in predator biomass with land use intensification at the study sites, but omitted soil living species. Our data shows that rainforest conversion causes a turnover of species in a major group of soil living predators, but that some species are resilient against the associated changes and that the overall number of ecological niches for specific guilds of predators may change little.

### 4.2. Changes of trophic niches of centipedes with conversion of rainforest

The resilience of centipedes to large scale changes of their environment is likely related to their ability to opportunistically switch to alternative prey. Stable isotope values of the species investigated were highly variable; in fact, the span of Δ^15^N values measured for each of the species *M*. cf. *stenoceps*, *Cryptops* sp. and *M*. cf. *enigmus* exceeded the value of 3.4 ‰, the mean increase in ^15^N from resource (prey) to consumers (predator) [34,35,63]. This confirms the hypothesis that in natural populations the prey spectrum of centipede species is variable with different individuals of the same species feeding on prey from different trophic levels. Presumably, individuals with low values mostly feed on decomposers, whereas those with high values regularly consume intra-guild prey, i.e. feed on other predators [64,65]. The conclusion that the diet of centipede species is highly variable is supported by the large range in Δ^13^C values of the studied species, indicating that the respective prey species utilized different basal resources. Thereby, variations of centipede stable isotope values were larger than variations of litter δ^13^C and δ^15^N values.

Conform to our second hypothesis, isotopic values of centipedes indicate substantial dietary shifts with the conversion of rainforest into agroforest / agricultural land-use systems. Tropical forest ecosystems provide ample amounts of leaf litter, which is a main basal resources for soil animal communities [59,66]. However, the soil of oil palm plantations in the study region is mostly bare of plant litter due to the common management practice of cutting and piling up palm fronds in rows between oil palms. Moreover, due to intensive application of fertilizers and the more open canopy as compared to rainforests and rubber plantations, oil palm plantations are characterized by a dense herb layer of mostly introduced weeds. Δ^13^C values of *M*. cf. *stenoceps*, a common species investigated across all transformation systems, were markedly lower in oil palm plantations as compared to rainforests, jungle rubber and rubber plantations. Δ^13^C values of a second species, *Cryptops* sp., followed the same trend. Including data from rare species indicates that this decrease applies to all centipede species. Stable isotope values therefore likely reflect a dietary shift from secondary decomposers, which characteristically are enriched in ^13^C [58,67] to a less ^13^C enriched trophic group of the food web. ^13^C values of ground living herbivores typically are lower than those of decomposers and similar to those of plants [68,69], suggesting that centipedes in oil palm plantations switched to a herbivore based diet. Overall, the shift in isotopic values corresponds to structural changes within the investigated habitats and suggests that in oil palm plantations generalist predators such as centipedes may compensate for low availability of decomposer prey by switching diets to herbivore species of the herb layer vegetation and potentially to herbivores of oil palm dropped to the soil by wind or heavy rainfall as documented for e.g., aphids in agroecosystems of the temperate zone [70].

## 5. Conclusions

The number of ecological niches of soil living centipedes in tropical rainforests remains similar even after substantial alteration of the aboveground habitat by conversion of rainforest to plantation systems. Changes in community composition suggest that this is due to euryoecious species replacing rainforest species, but also to the capability of some rainforest species to acclimate to altered conditions in replacement ecosystems by utilizing alternative prey. Identifying these alternative prey groups and developing effective measures to foster generalist predator populations in plantation systems may help in controlling insect pest species thereby contributing to the sustainable management of oil palm and other tropical monoculture plantation systems. Further, conservation measures are needed. A number of rainforest species likely cannot acclimate to conditions in converted systems and are replaced by more common species, which may result in the loss of unique evolutionary lines. The potential loss or weakening of associated ecological functions needs closer investigation. Investigating food web interactions using state-of-the-art techniques such as stable isotope, fatty acid and molecular gut content analysis is key for understanding how changes in community composition alters ecosystem functioning.

## Supporting information

S1 Table Regression coefficients and coefficient of determination (R^2^) of body size—dry weight relationships in two centipede groups of different body shape. N, number of replicates; L, body length; W, body width. Regression coefficients apply to the relation y = a(x)^b^, where y is dry weight (mg) and x is body length (L), body length times body width (L x W) or cylindrical volume (V = π(W/2)^2^L), respectively.
** p < 0.01, *** p < 0.001.
(DOCX)

S2 Table ANOVA results from generalized linear mixed effects models testing the effect of forest conversion on abundance, biomass and species richness of centipedes. Results are given for “intensity” models testing if conversion effects follow a linear trend from rainforest (F) to jungle rubber (J) to rubber (R) to oil palm plantations (O). Additionally, results are given for planned comparisons between conversion systems;* p < 0.05.
(DOCX)

S3 Table Summary table from Canonical Correspondence Analysis (CCA) investigating environmental variables structuring centipede communities. Explanatory variables (S4 Table) account for 33.4% of the variation, adjusted explained variation is 13.1%. Permutation Test results: Axis 1 pseudo-F = 3.5, p = 0.108; Fall axes pseudo-F = 1.6, p = 0.03.
(DOCX)

S4 Table Term effects of Canonical Correspondence Analysis (CCA) investigating correlations between abundances of centipede species and environmental variables. (DOCX)

S5 Table Abundances (ind./m^2^) of centipede species on research plots representing four different rainforest conversion systems in two study regions. (DOCX)

S6 Table List of centipede species including full taxonomic name and authority, family affiliation, number of replicates (stable isotope ratios) and mean δ^13^C and δ^15^N values (± SD). Abbr., abbreviation used in figures.
(DOCX)

S7 Table Stable isotope values of litter material sampled in different conversion systems. Number of replicates and mean δ^13^C and δ^15^N values (± SD).
(DOCX)

S1 Fig Relationship of body cross sectional area (length x width) to dry weight in two centipede groups of different body shape. (a) Geophilomorpha, (b) Cryptopidae and Henicopidae. Data were log-transformed to reduce heteroscedasticity and to linearize the size-weight relationship. Regression equations are additionally given back transformed to the power function. Coefficients of determination (R^2^-values) are given for both regressions, both regressions were highly significant (p < 0.001).
(DOCX)

S2 Fig Species accumulation curve showing the increase of centipede (Chilopoda) species with number of study sites. Means (' SD) based on 100 permutations. Dashed blue lines indicate bootstrap, first order jackknife and Chao extrapolations of the species pool in the study region [46].
(DOCX)

S3 Fig Discriminant function analysis of centipede (Chilopoda) species in rainforests (F), jungle rubber (J), rubber (R) and oil palm (O). Range ellipses for the discriminants are given at the 95% confidence level.
(DOCX)
