# Supplementary material for: Trophic niches, diversity and community composition of invertebrate top predators (Chilopoda) as affected by conversion of tropical lowland rainforest in Sumatra (Indonesia)
Source: PLoS One. 2017 Aug 1;12(8):e0180915. doi: 10.1371/journal.pone.0180915 (PMC5538669; doi:10.1371/journal.pone.0180915)
Supplement: S1 Table — N, number of replicates; L, body length; W, body width. Regression coefficients apply to the relation y = a(x)b, where y is dry weight (mg) and x is body length (L), body length times body width (L x W) or cylindrical volume (V = π(W/2)2L), respectively. ** p < 0.01, *** p < 0.001. (DOCX) [file pone.0180915.s001.docx]

**S1 Table. Regression coefficients and coefficient of determination (R^2^) of body size - dry weight relationships in two centipede groups of different body shape.**

| **Group** | **N** | **Size range** | **Size parameter** | **a** | **b** | **R^2^** |
| --- | --- | --- | --- | --- | --- | --- |
| Geophilomorpha | 49 | L : 3.9 - 28.0 mm, W: 0.12 - 0.9 mm | L | 0.0001 | 3.226 | 0.79*** |
|  |  |  | L x W | 0.019 | 1.625 | 0.83*** |
|  |  |  | V | 0.159 | 1.057 | 0.81*** |
| Cryptopidae, Henicopidae | 17 | L : 3.2 - 12.0 mm, W: 0.18 - 0.73 mm | L | 0.0007 | 2.526 | 0.46** |
|  |  |  | L x W | 0.019 | 1.496 | 0.62*** |
|  |  |  | V | 0.099 | 0.926 | 0.60*** |

N, number of replicates; L, body length; W, body width. Regression coefficients apply to the relation y = a(x)^b^, where y is dry weight (mg) and x is body length (L), body length times body width (L x W) or cylindrical volume (V = π(W/2)^2^L), respectively.

** p < 0.01, *** p < 0.001.
