# Supplementary material for: Trophic niches, diversity and community composition of invertebrate top predators (Chilopoda) as affected by conversion of tropical lowland rainforest in Sumatra (Indonesia)
Source: PLoS One. 2017 Aug 1;12(8):e0180915. doi: 10.1371/journal.pone.0180915 (PMC5538669; doi:10.1371/journal.pone.0180915)
Supplement: S2 Table — Results are given for “intensity” models testing if conversion effects follow a linear trend from rainforest (F) to jungle rubber (J) to rubber (R) to oil palm plantations (O). Additionally, results are given for planned comparisons between conversion systems;* p < 0.05. (DOCX) [file pone.0180915.s002.docx]

**S2 Table. ANOVA results from generalized linear mixed effects models testing the effect of forest conversion on abundance, biomass and species richness of centipedes.**

| Contrast | Abundance | Biomass | Species richness |
| --- | --- | --- | --- |
| „Intensity“ | F_1,30_ = 0.98 | F_1,30_ = 0.02 | F_1,30_ = 0.79 |
| F vs. J | F_1,30_ = 0.26 | F_1,30_ = 0.75 | F_1,30_ = 0.22 |
| F vs. R | **F_1,30_ = 5.75*** | F_1,30_ = 1.56 | F_1,30_ = 1.99 |
| F vs. O | F_1,30_ = 0.22 | F_1,30_ = 0.00 | F_1,30_ = 1.21 |
| J vs. R | F_1,30_ = 3.15 | F_1,30_ = 0.13 | F_1,30_ = 0.89 |
| J vs. O | F_1,30_ = 0.00 | F_1,30_ = 0.71 | F_1,30_ = 0.39 |
| R vs. O | F_1,30_ = 3.34 | F_1,30_ = 1.50 | F_1,30_ = 0.10 |

Results are given for “intensity” models testing if conversion effects follow a linear trend from rainforest (F), over jungle rubber (J), to rubber- (R) and oil palm plantation (O). Additionally, results are given for planned comparisons between conversion systems.

* p < 0.05.
