# Supplementary material for: Trophic niches, diversity and community composition of invertebrate top predators (Chilopoda) as affected by conversion of tropical lowland rainforest in Sumatra (Indonesia)
Source: PLoS One. 2017 Aug 1;12(8):e0180915. doi: 10.1371/journal.pone.0180915 (PMC5538669; doi:10.1371/journal.pone.0180915)
Supplement: S3 Table — Explanatory variables (S4 Table) account for 33.4% of the variation, adjusted explained variation is 13.1%. Permutation Test results: Axis 1 pseudo-F = 3.5, p = 0.108; Fall axes pseudo-F = 1.6, p = 0.03. (DOCX) [file pone.0180915.s003.docx]

**S3 Table. Summary table from canonical correspondence analysis (CCA) investigating environmental variables structuring centipede communities.**

| Statistic | Axis 1 | Axis 2 | Axis 3 | Axis 4 |
| --- | --- | --- | --- | --- |
| Eigenvalues | 0.37 | 0.27 | 0.18 | 0.07 |
| Explained variation (cumulative) [%] | 13.18 | 22.69 | 29.16 | 31.72 |
| Pseudo-canonical correlation | 0.86 | 0.78 | 0.65 | 0.52 |
| Explained fitted variation (cumulative) [%] | 39.49 | 67.96 | 87.35 | 95.04 |
| Pseudo-canonical correlation (suppl.) | 0.55 | 0.62 | 0.41 | 0.16 |

Explanatory variables (S4 Table) account for 33.4% of the variation, adjusted explained variation is 13.1%. Permutation Test results: Axis 1 pseudo-F = 3.5, p = 0.108; all axes pseudo-F = 1.6, p = 0.03.
