# Supplementary material for: Trophic niches, diversity and community composition of invertebrate top predators (Chilopoda) as affected by conversion of tropical lowland rainforest in Sumatra (Indonesia)
Source: PLoS One. 2017 Aug 1;12(8):e0180915. doi: 10.1371/journal.pone.0180915 (PMC5538669; doi:10.1371/journal.pone.0180915)
Supplement: S4 Table — (DOCX) [file pone.0180915.s004.docx]

**S4 Table. Term effects of canonical correspondence analysis (CCA) investigating correlations between abundances of centipede species and environmental variables.**

|  | Env. constraint | Expl. variation [%] | pseudo-F | P |
| --- | --- | --- | --- | --- |
| Simple Effects | pH litter | 9.8 | 3.1 | **0.004** |
|  | Amount litter | 8.8 | 2.8 | **0.048** |
|  | pH soil | 8.2 | 2.6 | 0.056 |
|  | C/N soil | 6.4 | 2.0 | 0.108 |
|  | C_mic_ litter | 4.7 | 1.4 | 0.188 |
|  | C_mic_ soil | 4.3 | 1.3 | 0.244 |
|  | C/N litter | 3.7 | 1.1 | 0.34 |
| Conditional Effects | pH litter | 9.8 | 3.1 | **0.002** |
|  | Amount litter | 6.4 | 2.1 | 0.076 |
|  | pH soil | 4.3 | 1.5 | 0.168 |
|  | C/N soil | 3.7 | 1.3 | 0.26 |
|  | C_mic_ litter | 2.8 | 1.0 | 0.374 |
|  | C_mic_ soil | 4.0 | 1.4 | 0.202 |
|  | C/N litter | 2.3 | 0.8 | 0.554 |
