# Supplementary material for: Trophic niches, diversity and community composition of invertebrate top predators (Chilopoda) as affected by conversion of tropical lowland rainforest in Sumatra (Indonesia)
Source: PLoS One. 2017 Aug 1;12(8):e0180915. doi: 10.1371/journal.pone.0180915 (PMC5538669; doi:10.1371/journal.pone.0180915)
Supplement: S5 Table — (DOCX) [file pone.0180915.s005.docx]

**S5 Table. Abundances (ind./m^2^) of centipede species on research plots representing four different rainforest conversion systems in two study regions.**

| **Region** | **Conversion system** | **Plot** | ***Cryptops* sp.** | ***Lamyctes* sp.** | ***Mecistocephalus* cf. *enigmus*** | ***Mecistocephalus* cf. *stenoceps*** | ***Mecistocephalus* cf. *verrucosus*** | ***Paracryptops* sp.** | **Schendylidae** | ***Sundageophilus bidentatus*** | ***Sundageophilus poriger*** | ***Tygarrup* cf. *javanicus*** | **undetermined Mecistocephalidae** | **undetermined Scolopendridae** |
| --- | --- | --- | --- | --- | --- | --- | --- | --- | --- | --- | --- | --- | --- | --- |
| Bukit Duabelas | Rainforest | BF1 | 13 | 13 | 0 | 0 | 0 | 0 | 0 | 13 | 0 | 0 | 0 | 13 |
|  |  | BF2 | 65 | 52 | 0 | 52 | 0 | 0 | 0 | 13 | 0 | 0 | 0 | 0 |
|  |  | BF3 | 65 | 0 | 13 | 52 | 0 | 0 | 0 | 91 | 0 | 0 | 13 | 0 |
|  |  | BF4 | 39 | 0 | 26 | 26 | 0 | 0 | 0 | 13 | 0 | 0 | 0 | 0 |
|  | Jungle rubber | BJ2 | 13 | 0 | 0 | 0 | 13 | 0 | 0 | 0 | 52 | 0 | 0 | 0 |
|  |  | BJ3 | 0 | 65 | 0 | 0 | 0 | 0 | 0 | 0 | 13 | 0 | 0 | 0 |
|  |  | BJ4 | 13 | 0 | 0 | 13 | 0 | 0 | 0 | 0 | 0 | 0 | 0 | 0 |
|  |  | BJ5 | 0 | 0 | 0 | 0 | 0 | 0 | 0 | 0 | 13 | 0 | 0 | 0 |
|  | Rubber | BR1 | 0 | 13 | 0 | 39 | 0 | 0 | 0 | 0 | 0 | 0 | 13 | 0 |
|  |  | BR2 | 0 | 0 | 0 | 0 | 0 | 0 | 0 | 0 | 0 | 0 | 0 | 0 |
|  |  | BR3 | 0 | 13 | 0 | 26 | 0 | 0 | 0 | 0 | 0 | 0 | 0 | 0 |
|  |  | BR4 | 0 | 13 | 0 | 0 | 0 | 0 | 0 | 0 | 0 | 0 | 0 | 0 |
|  | Oil palm | BO2 | 0 | 0 | 0 | 78 | 0 | 0 | 0 | 0 | 0 | 0 | 0 | 0 |
|  |  | BO3 | 26 | 78 | 0 | 39 | 0 | 0 | 0 | 0 | 0 | 0 | 0 | 0 |
|  |  | BO4 | 0 | 0 | 0 | 26 | 0 | 0 | 0 | 13 | 0 | 0 | 0 | 0 |
|  |  | BO5 | 0 | 0 | 0 | 39 | 0 | 0 | 65 | 0 | 0 | 0 | 0 | 0 |
| Harapan | Rainforest | HF1 | 39 | 0 | 0 | 78 | 0 | 0 | 0 | 0 | 0 | 13 | 0 | 0 |
|  |  | HF2 | 0 | 0 | 0 | 0 | 0 | 0 | 0 | 0 | 0 | 0 | 0 | 13 |
|  |  | HF3 | 0 | 0 | 0 | 13 | 0 | 0 | 0 | 26 | 0 | 0 | 0 | 0 |
|  |  | HF4 | 39 | 0 | 0 | 78 | 0 | 0 | 0 | 0 | 0 | 0 | 0 | 0 |
|  | Jungle rubber | HJ1 | 0 | 0 | 13 | 39 | 0 | 13 | 0 | 0 | 0 | 0 | 0 | 0 |
|  |  | HJ2 | 26 | 0 | 13 | 78 | 26 | 0 | 0 | 13 | 13 | 0 | 0 | 0 |
|  |  | HJ3 | 0 | 13 | 0 | 104 | 0 | 0 | 0 | 0 | 0 | 0 | 0 | 0 |
|  |  | HJ4 | 0 | 39 | 0 | 13 | 0 | 0 | 0 | 26 | 0 | 0 | 0 | 0 |
|  | Rubber | HR1 | 0 | 52 | 0 | 13 | 0 | 0 | 0 | 0 | 0 | 0 | 0 | 0 |
|  |  | HR2 | 13 | 39 | 0 | 13 | 0 | 0 | 0 | 0 | 0 | 13 | 0 | 0 |
|  |  | HR3 | 0 | 26 | 0 | 13 | 0 | 0 | 0 | 0 | 0 | 0 | 0 | 0 |
|  |  | HR4 | 13 | 13 | 0 | 0 | 0 | 0 | 0 | 0 | 0 | 0 | 0 | 0 |
|  | Oil palm | HO1 | 0 | 52 | 0 | 13 | 0 | 0 | 0 | 0 | 0 | 0 | 0 | 0 |
|  |  | HO2 | 13 | 0 | 0 | 13 | 0 | 0 | 0 | 0 | 0 | 0 | 0 | 0 |
|  |  | HO3 | 39 | 0 | 0 | 13 | 0 | 0 | 0 | 0 | 13 | 0 | 0 | 0 |
|  |  | HO4 | 13 | 0 | 0 | 13 | 0 | 0 | 26 | 0 | 0 | 0 | 0 | 0 |
