# Supplementary material for: Trophic niches, diversity and community composition of invertebrate top predators (Chilopoda) as affected by conversion of tropical lowland rainforest in Sumatra (Indonesia)
Source: PLoS One. 2017 Aug 1;12(8):e0180915. doi: 10.1371/journal.pone.0180915 (PMC5538669; doi:10.1371/journal.pone.0180915)
Supplement: S6 Table — Abbr., abbreviation used in figures. (DOCX) [file pone.0180915.s006.docx]

**S6 Table. List of centipede species including full taxonomic name and authority, family affiliation, number of replicates (stable isotope ratios) and mean δ^13^C and δ^15^N values (± SD).**

| **Abbr.** | **Full name** | **Family** | **N** | **δ^13^C** | **δ^15^N** |
| --- | --- | --- | --- | --- | --- |
| Cryp_sp | *Cryptops sp.* Leach, 1814 | Cryptopidae | 14 | -25.50 ± 0.96 | 6.55 ± 1.31 |
| Lam_sp | *Lamyctes sp.* Meinert, 1868 | Henicopidae | 3 | -24.83 ± 1.58 | 6.95 ± 1.25 |
| Mec_eni | *Mecistocephalus* cf. *enigmus* Chamberlin, 1944 | Mecistocephalidae | 2 | -25.56 ± 0.63 | 7.65 ± 1.79 |
| Mec_ste | *Mecistocephalus* cf. *stenoceps* Chamberlin, 1944 | Mecistocephalidae | 28 | -25.56 ± 1.24 | 7.71 ± 0.96 |
| Mec_ver | *Mecistocephalus* cf. *verrucosus* Verhoeff, *1937* | Mecistocephalidae | 2 | -25.24 ± 1.17 | 8.38 ± 1.98 |
| Sche_sp | undetermined Schendylidae | Schendylidae | 3 | -27.33 ± 0.53 | 5.97 ± 1.13 |
| Sco_sp | undetermined Scolopendridae | Scolopendridae | 1 | -25.47 | 7.45 |
| Sun_bid | *Sundageophilus bidentatus* Bonato, 2016 | Geophilidae | 6 | -24.93 ± 0.31 | 7.5 ± 0.76 |
| Sun_por | *Sundageophilus poriger* Bonato, 2016 | Geophilidae | 5 | -25.86 ± 0.7 | 7.16 ± 0.62 |
| Tyg_jav | *Tygarrup* cf. *javanicus* Attems, 1929 | Mecistocephalidae | 1 | -26.49 | 5.86 |

Abbr., abbreviation used in figures.
