# Supplementary material for: Trophic niches, diversity and community composition of invertebrate top predators (Chilopoda) as affected by conversion of tropical lowland rainforest in Sumatra (Indonesia)
Source: PLoS One. 2017 Aug 1;12(8):e0180915. doi: 10.1371/journal.pone.0180915 (PMC5538669; doi:10.1371/journal.pone.0180915)
Supplement: S7 Table — Number of replicates and mean δ13C and δ15N values (± SD). (DOCX) [file pone.0180915.s007.docx]

**S7 Table. Stable isotope values of litter material sampled in different conversion systems.**

| **Conversion system** | **N** | **δ^13^C** | **δ^15^N** |
| --- | --- | --- | --- |
| Rainforest | 19 | -30.94 ± 1.23 | 0.12 ± 0.56 |
| Jungle rubber | 14 | -30.52 ± 0.8 | -0.98 ± 1.43 |
| Rubber | 17 | -29.36 ± 0.71 | -0.45 ± 1.78 |
| Oil Palm | 23 | -30.11 ± 1.43 | 0.64 ± 0.94 |

Number of replicates and mean δ^13^C and δ^15^N values (± SD).
