# Supplementary material for: Trophic niches, diversity and community composition of invertebrate top predators (Chilopoda) as affected by conversion of tropical lowland rainforest in Sumatra (Indonesia)
Source: PLoS One. 2017 Aug 1;12(8):e0180915. doi: 10.1371/journal.pone.0180915 (PMC5538669; doi:10.1371/journal.pone.0180915)
Supplement: S1 Fig — (a) Geophilomorpha, (b) Cryptopidae and Henicopidae. Data were log-transformed to reduce heteroscedasticity and to linearize the size-weight relationship. Regression equations are additionally given back transformed to the power function. Coefficients of determination (R2-values) are given for both regressions, both regressions were highly significant (p < 0.001). (DOCX) [file pone.0180915.s008.docx]

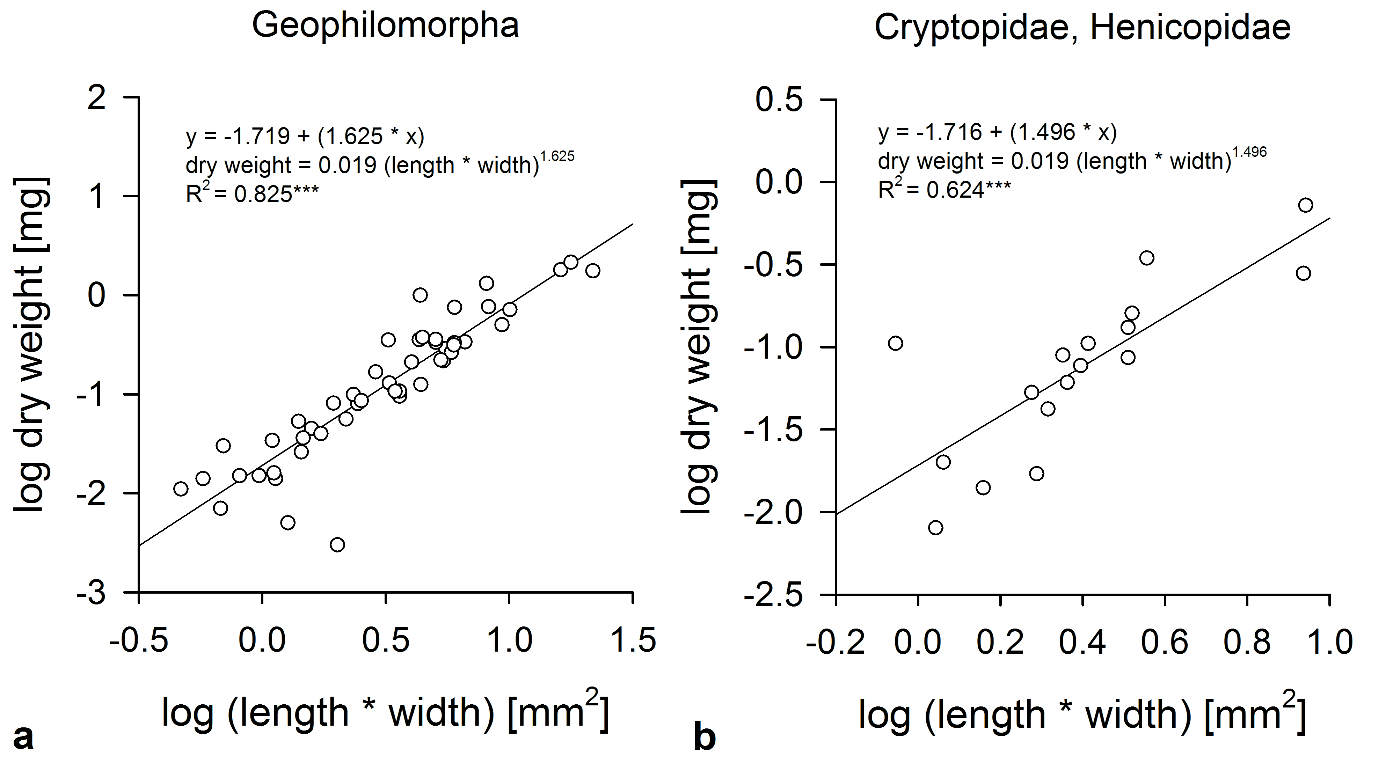


**S1 Fig. Relationship of body cross sectional area (length x width) to dry weight in two centipede groups of different body shape.**

(a) Geophilomorpha, (b) Cryptopidae and Henicopidae. Data were log-transformed to reduce heteroscedasticity and to linearize the size-weight relationship. Regression equations are additionally given back transformed to the power function. Coefficients of determination (R^2^-values) are given for both regression, both regressions were highly significant (p < 0.001).
