# Supplementary material for: Trophic niches, diversity and community composition of invertebrate top predators (Chilopoda) as affected by conversion of tropical lowland rainforest in Sumatra (Indonesia)
Source: PLoS One. 2017 Aug 1;12(8):e0180915. doi: 10.1371/journal.pone.0180915 (PMC5538669; doi:10.1371/journal.pone.0180915)
Supplement: S2 Fig — Means (' SD) based on 100 permutations. Dashed blue lines indicate bootstrap, first order jackknife and Chao extrapolations of the species pool in the study region [46]. (DOCX) [file pone.0180915.s009.docx]

**
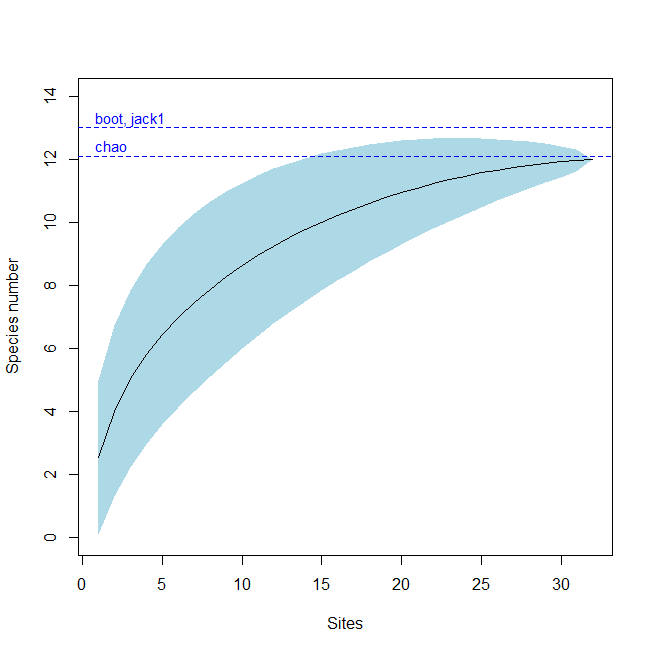
**

**S2 Fig.** **Species accumulation curve showing the increase of centipede (Chilopoda) species with number of study sites.**

Means (± SD) based on 100 permutations. Dashed blue lines indicate bootstrap, first order jackknife and Chao extrapolations of the species pool in the study region [46].
