# Supplementary material for: Trophic niches, diversity and community composition of invertebrate top predators (Chilopoda) as affected by conversion of tropical lowland rainforest in Sumatra (Indonesia)
Source: PLoS One. 2017 Aug 1;12(8):e0180915. doi: 10.1371/journal.pone.0180915 (PMC5538669; doi:10.1371/journal.pone.0180915)
Supplement: S3 Fig — Range ellipses for the discriminants are given at the 95% confidence level. (DOCX) [file pone.0180915.s010.docx]

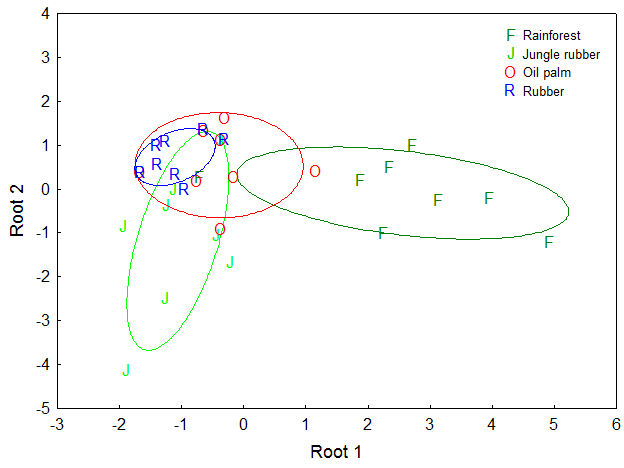


**S3 Fig. Discriminant function analysis of centipede (Chilopoda) species in rainforests (F), jungle rubber (J), rubber (R) and oil palm (O).**

Range ellipses for the discriminants are given at the 95% confidence level.
